# Extracorporeal shock wave therapy for bone marrow edema syndrome in patients with osteonecrosis of the femoral head: a retrospective cohort study

**DOI:** 10.1186/s13018-020-02159-7

**Published:** 2021-01-07

**Authors:** Wenyi Zhao, Yuan Gao, Shouxiang Zhang, Zhang Liu, Lin He, Dahong Zhang, Wei Li, Qinggang Meng

**Affiliations:** 1 Harbin Medical University, Harbin, China; 2 Bone and Joint Surgery Department, First Hospital of Suihua City, Suihua, China; 3 Bone and Joint Surgery Department, Pinghu Hospital Affiliated to Shenzhen University, Shenzhen, China; 4 Bone and Joint Surgery Department, Harbin City Hospital No. 1 Affiliated to Harbin Technical University, Harbin, China

**Keywords:** Extracorporeal shockwave therapy, Osteonecrosis of the femoral head, Bone marrow edema syndrome, Harris hip score, Charnley score

## Abstract

**Background:**

There is now ample evidence suggesting that extracorporeal shock wave therapy (ESWT) can improve hip mobility and reduce pain in patients with osteonecrosis of the femoral head (ONFH). The ability of ESWT to cure bone marrow edema syndrome (BMES) in patients with ONFH, 12 weeks after the initial course of ESWT, needs to be verified further and more relevant clinical research-based evidence should be consolidated. This study aimed to evaluate the efficacy of ESWT for BMES caused by ONFH.

**Methods:**

This retrospective cohort study included 67 patients with BMES caused by ONFH who were participating in a rehabilitation program as outpatients. Before and after ESWT, the area of femoral bone marrow edema was evaluated by magnetic resonance imaging (MRI), and the Harris score and Charnley score were evaluated as hip pain and function indicators.

**Results:**

After ESWT, MRI revealed that the area of bone marrow edema decreased from 984.6 ± 433.2 mm^2^ to 189.7 ± 214.4 mm^2^ (*P* < 0.0001). The Harris score increased from 42.2 ± 9.1 to 77.7 ± 10.8 points (*P* < 0.0001). The Charnley score increased from 7.3 ± 1.4 to 12.0 ± 1.7 (*P* < 0.0001). ESWT was effective in treating BMES in 98.5% of the cases.

**Conclusions:**

This study demonstrated that ESWT can effectively treat BMES caused by ONFH and can aid in pain relief and functional recovery in patients with ONFH. Thus, ESWT should be included in the classic physical therapy regimen for patients with ONFH and BMES.

## Background

Numerous studies have proven that extracorporeal shock wave therapy (ESWT) has a positive effect on the treatment of musculoskeletal diseases [1, 2]. However, there are few reports on its effect for the treatment of osteonecrosis of the femoral head (ONFH) associated with bone marrow edema syndrome, which may have various causes. ONFH is caused by a variety of disorders of local blood circulation in the femoral head, resulting in the death of the active components of the bone (bone cells, bone marrow cells). This eventually leads to the destruction of the trabecular bone in the femoral head, the collapse of the femoral head, and the loss of joint function [3]. Bone marrow edema syndrome (BMES) is an increase in interstitial fluid in the space between bone marrows. BMES is a common concomitant sign in the development of ONFH. The clinical symptoms are characterized by increasing hip pain, which usually resolves spontaneously within approximately 6 to 9 months [4]. Compared with joint effusions in ONFH, BMES is more related to pain [5]. Studies have shown that the hip pain symptom classification of ONFH patients positively correlates with the degree and area ratio of bone marrow edema [6]. Magnetic resonance imaging (MRI), T1-weighted images, and short tau inversion recovery and T2-weighted images showed typical characteristics, low signal, and high signal, respectively [7]. In mild BMES, bone marrow edema only appears around the necrotic area. If the BMES is aggravated, the area of bone marrow edema can expand to the femoral neck; in severe BMES, it expands below the femoral trochanter. The histological findings of the corresponding area of BMES are edema in the bone marrow interstitium, amorphous exudate between fat cells, fibrous tissue hyperplasia, and chronic inflammatory cell infiltration [8]. Although many bone lacunae are empty, the trabecular bone remains active, and there is no sign of necrosis in the trabecular bone and bone marrow tissue, showing that the area of bone marrow edema is not an extension of the necrosis range [9]. Pathological analysis of the BMES area showed a widened trabecular bone space filled with significant amounts of edema fluid, fat necrosis, continuous trabecular bone, the survival of bone cells, reactive bone formation, and no bone resorption of progressive osteoclasts. The increase of bone-like and the decrease of hydroxyapatite content can explain the decrease of bone density on a radiograph.

The survival of trabecular bone and active bone formation indicate a strong regeneration ability [10]. There are still many controversies about the pathogenesis and pathological mechanism of BMES, and its relationship with ONFH. At present, there are mainly three views as follows: (1) BMES is a congestive edema caused by early repair response in the area of osteonecrosis [11]; (2) BMES is caused by ONFH intraosseous pressure caused by femoral vein reflux disturbance, capillary endothelial cell damage, increased exudation, and increased bone marrow interstitial water content [12]; and (3) mechanical stress is the cause of BMES [13]. In summary, the cause of BMES secondary to ONFH is more complicated, and it is difficult for a single pathological factor to provide a reasonable and comprehensive explanation.

Extracorporeal shock wave therapy (ESWT) is non-invasive, safe, and effective. It is widely used in the clinical treatment of musculoskeletal diseases [14]. A shock wave is an acoustic wave with mechanical properties that produces energy through the rapid or extreme compression of a medium caused by vibration and high-speed motion. Its physical characteristics include mechanical effect, cavitation effect, thermal effect, and biological effects, which include tissue repair and reconstruction [15], tissue adhesion release [16], dilation of blood vessels and angiogenesis [17], analgesia and nerve ending blockade [18], and inflammation and infection control [19]. While core decompression is the gold standard, the quality of outcomes is slightly debatable, and therefore, the choice of treatment should depend on the state of disease progression. A recent meta-analysis comparing core decompression with other joint preserving treatments for osteonecrosis of the femoral head showed that core decompression was not any better than that of conservative treatment, and therefore, conservative treatment should be given priority [20]. ESWT in particular has a strong analgesic ability; for example, the treatment can significantly reduce the VAS score of patients with plantar fasciitis [2]. ESWT has been used to treat BMES caused by ONFH [21]. Therefore, we used focused shock waves to treat BMES of the femoral head and observed its clinical efficacy to provide medical evidence for clinical ESWT treatment of BMES.

## Methods

### Participants and design

**Fig. 1:**
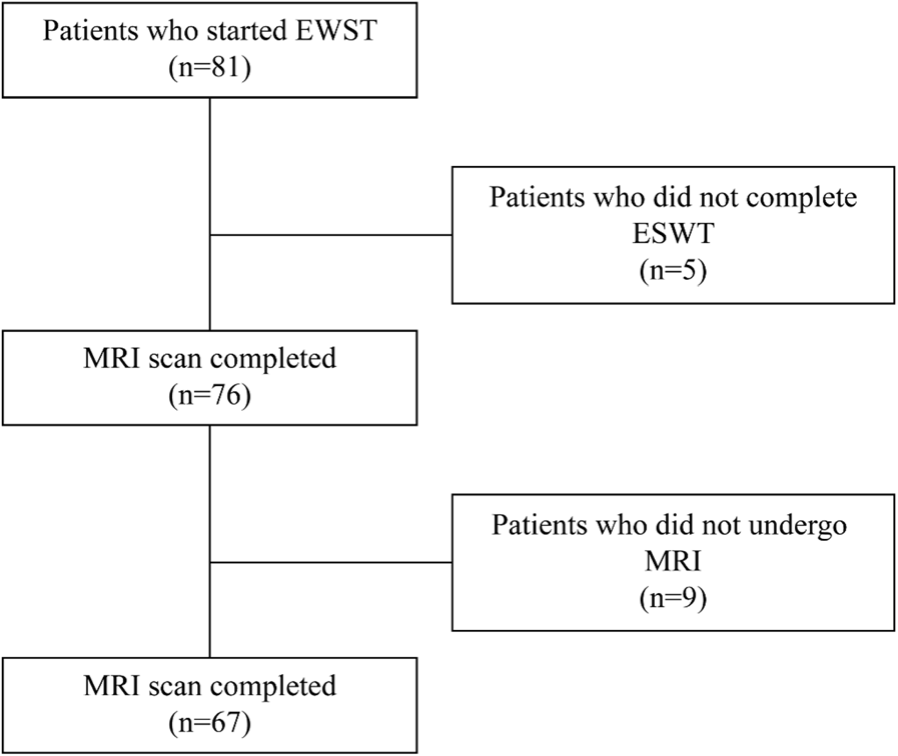
Flow chart diagram

This retrospective cohort study was conducted at the Harbin City Hospital No. 1 Center. It evaluated the efficacy of ESWT on BMES of ONFH. A total of 67 patients (24 men and 43 women), aged 25–73 years (average age, 48.6 ± 11.2 years), were diagnosed with ONFH accompanied by BMES and were treated with ESWT after outpatient diagnosis (Fig. 1). The inclusion criteria were fulfillment of the diagnostic criteria for femoral head necrosis according to the Association Research Circulation Osseous (ARCO) classification system; stage I, II, III, or IV ONFH and age of 18–80 years. The exclusion criteria were pregnancy, a tumor lesion located at the femoral head as the cause of ONFH, acute soft tissue infection, or skin disruption in the topically treated area. All participants signed an informed consent form, which was approved by the ethics committee of Harbin First Hospital.

### Experimental procedure

#### Experimental design

The study included patients who were diagnosed with ONFH accompanied BMES in our outpatient clinic from November 2018 to June 2020 and had completed 12 ESWT treatment cycles. According to the medical history, the etiologies of ONFH were obtained, and the patients were divided into four groups: Hormonal agent, Alcohol, Trauma, and Idiopathic groups. Patients underwent MRI scanning before and after ESWT treatment and completed Harris hip score (HHS) and Charnley score.

Effective treatment was defined as a 60% reduction in the area of bone marrow edema and significant relief of the patient’s hip symptoms; complete disappearance of bone marrow edema was defined as being cured.

#### Sample size calculation

The sample size was estimated to be 4 recipients, in line with the qualitative response parameters (*α*= 0.05, double; power of test 1- *β* = 0.9; power = 0.9923). This retrospective cohort study included 67 patients with BMES caused by ONFH, more than the sample size number.

#### Diagnostic methods and standards

Two joint surgeons diagnosed femoral head necrosis based on the patient’s medical history, symptoms, signs, and imaging findings; scored the degree of pain in the affected hip according to the pain score item in the HHS and Charnley score; and classified the affected hip in accordance with the ARCO International Staging Standard for necrosis staging [22]. Two radiologists with more than 5 years of working experience with musculoskeletal disorders subsequently diagnosed and classified the imaging data according to the diagnostic criteria of bone marrow edema; they then calculated the area of bone marrow edema. The hip joint was scanned with high-resolution MRI equipment (Discovery MR750w 3.0 T, GE Healthcare, Boston, MA, USA); the slice thickness at the hip joint was 0.2 mm.

MRI diagnostic criteria for bone marrow edema were as follows [23]: a T1-weighted image below the fat signal in the medullary cavity, a diffuse high-signal image outside the lesion on the T2-weighted image, and a clear high signal on the T2-fat suppression image.

#### Personalized ESWT

**Fig. 2:**
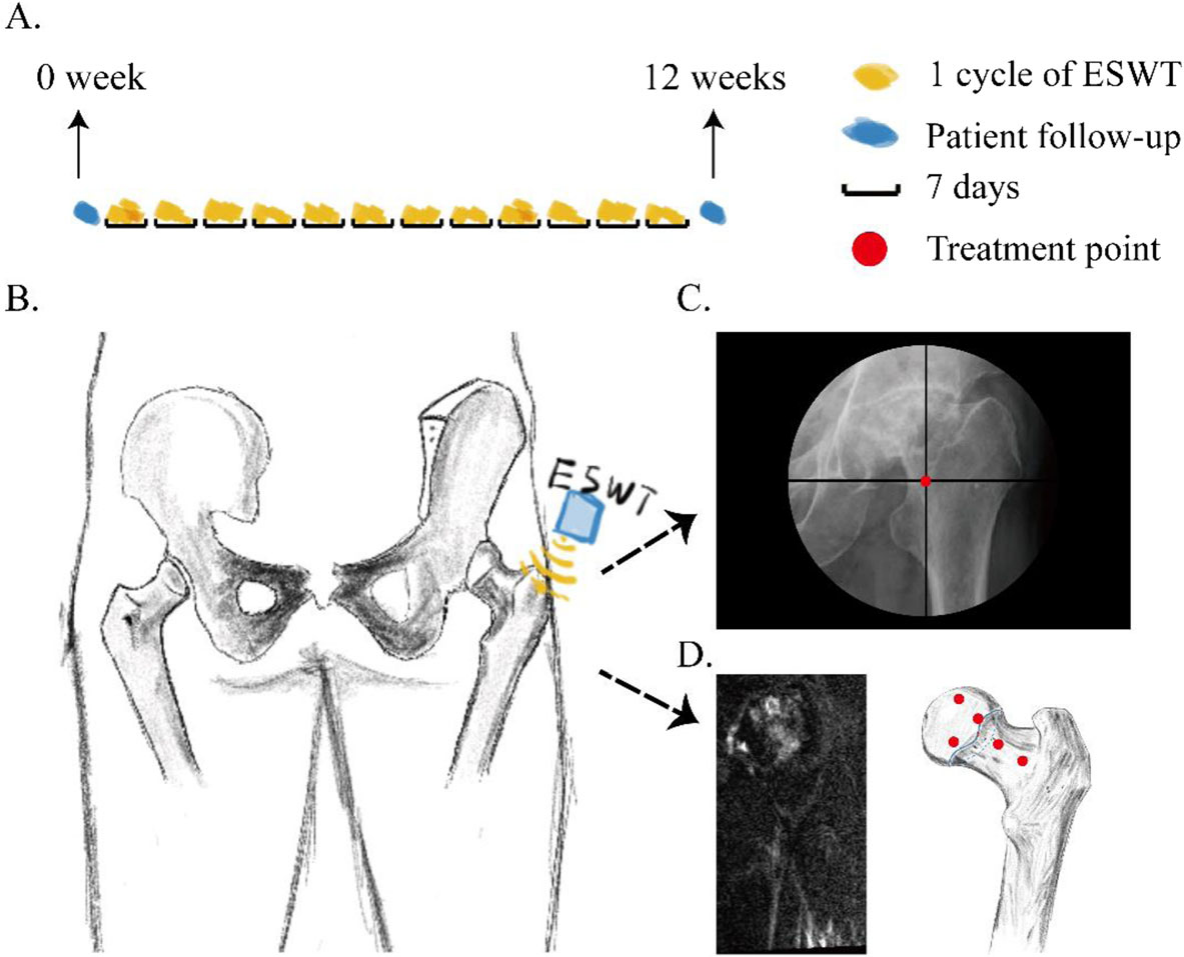
Personalized extracorporeal shockwave therapy (ESWT). **a** Technical solution. **b** Treatment diagram. **c** ESWT with the guidance of the C-arm. **d** The treatment point was selected according to the magnetic resonance imaging findings of bone marrow edema syndrome

ESWT was performed using the following procedure: Starting with 0.25 mJ/mm^2^, shock wave energy was gradually increased to 0.40 mJ/mm^2^ over the course of three cycles of ESWT depending on the patient’s sensitivity to pain. Each treatment point was pulsed 700 times, and the treatment was performed every 3 days, with two treatments per cycle, resulting in 12 consecutive cycles of ESWT treatment (Fig. 2a). The application of focused ESWT equipment (XYS.GU-6X; XinYuanSu Inc., Shenzhen, China) was conducted without anesthesia by the same experienced orthopedist, using ESWT under the guidance of the C-arm, in all the patients (Fig. 2b, c). According to the area of bone marrow edema, 3–6 treatment points were selected on each side of the femur, and important nerves and blood vessels were avoided (Fig. 2d). Any adverse events occurring during ESWT were recorded.

#### General treatment

Generally, treatment included quitting smoking and drinking, and strictly limiting femoral weight-bearing by using crutches and other appliances. Patients and their families received training on necessary medical knowledge so that they could complete the above treatment plan together.

#### Hip function evaluation

To reduce the bias of hip evaluation, the hip function was assessed both using the HHS [24] and the Charnley score [25]. HHS emphasizes the importance of pain and function; the evaluation method is a comprehensive score of the four indicators of pain, function, deformity, and joint mobility; and the evaluation result is represented by a single total score. The Charnley score includes three items: pain, walking ability, and activity. The Charnley score is simple and convenient, and the grade score is easy to remember and use. Grading according to walking ability can aid in evaluating unilateral and bilateral patients. Hip function evaluation is particularly important in rehabilitation guidance and enables timely detection of hidden hip problems. All these evaluations are based on a patient-centered evaluation system.

#### Statistical analysis

We analyzed the data of all patients with MRI data on the BMES of the femoral head, i.e., patients with valid follow-up data for 12 weeks after ESWT; there was no attribution to missing data. The Student’s paired *t* test was used to compare HHS score, Charnley score, and BMES area before and after ESWT; a 60% reduction in BMES area was classified as a successful treatment, while the progression of BMES area was classified as treatment failure.

The patient data were analyzed using GraphPad Prism version 7.0 (GraphPad Software Inc., San Diego, CA, USA). A *P* value < 0.05 was considered statistically significant. The difference test of single group mean was used to calculate the sample size using power analysis and Sample Size 15.0.5 software (SAS Institute Inc., USA).

According to the literature, BMES symptoms had significantly improved at 12 weeks in the ESWT group, and after 24 weeks in the untreated group, on average [5]; therefore, the time for significant improvement of BMES symptoms was taken as the endpoint of this study, while the untreated, natural process was considered the baseline [26].

## Results

After the patients were included in the study, no data was lost. The subjects’ demographic and clinical characteristics at baseline are shown in Table 1.

**Table 1:** Demographic and clinical characteristics at baseline

Variable	ESWT group
Age, years	48.6 ± 11.2 (range, 25–73)
Male/female, *n*	24/43
ARCO stage, *n*	
I	10
II	33
III	24
Etiology, *n*	
Hormonal agent	25
Alcohol	11
Trauma	23
Idiopathic	8
Duration of follow-up, months	3

*ESWT* extracorporeal shockwave therapy, *ARCO* Association Research Circulation Osseous

Table 2 shows the original data, including the mean and standard deviation of all dependent variables before and after the test. Statistical significance was set to *P* < 0.05 (two-sided) for all analyses.

**Table 2:** Change in hip function evaluation score after extracorporeal shockwave therapy

ESWT	Before (0 week)	After (12 weeks)	*P* value
HHS	42.2 ± 9.1	77.7 ± 10.8	< 0.0001
Charnley score	7.3 ± 1.4	12.0 ± 1.7	< 0.0001
Area of bone marrow edema, mm^2^	984.6 ± 433.2	189.7 ± 214.4	< 0.0001

*ESWT* extracorporeal shockwave therapy, *HHS* Harris hip score

**Fig. 3:**
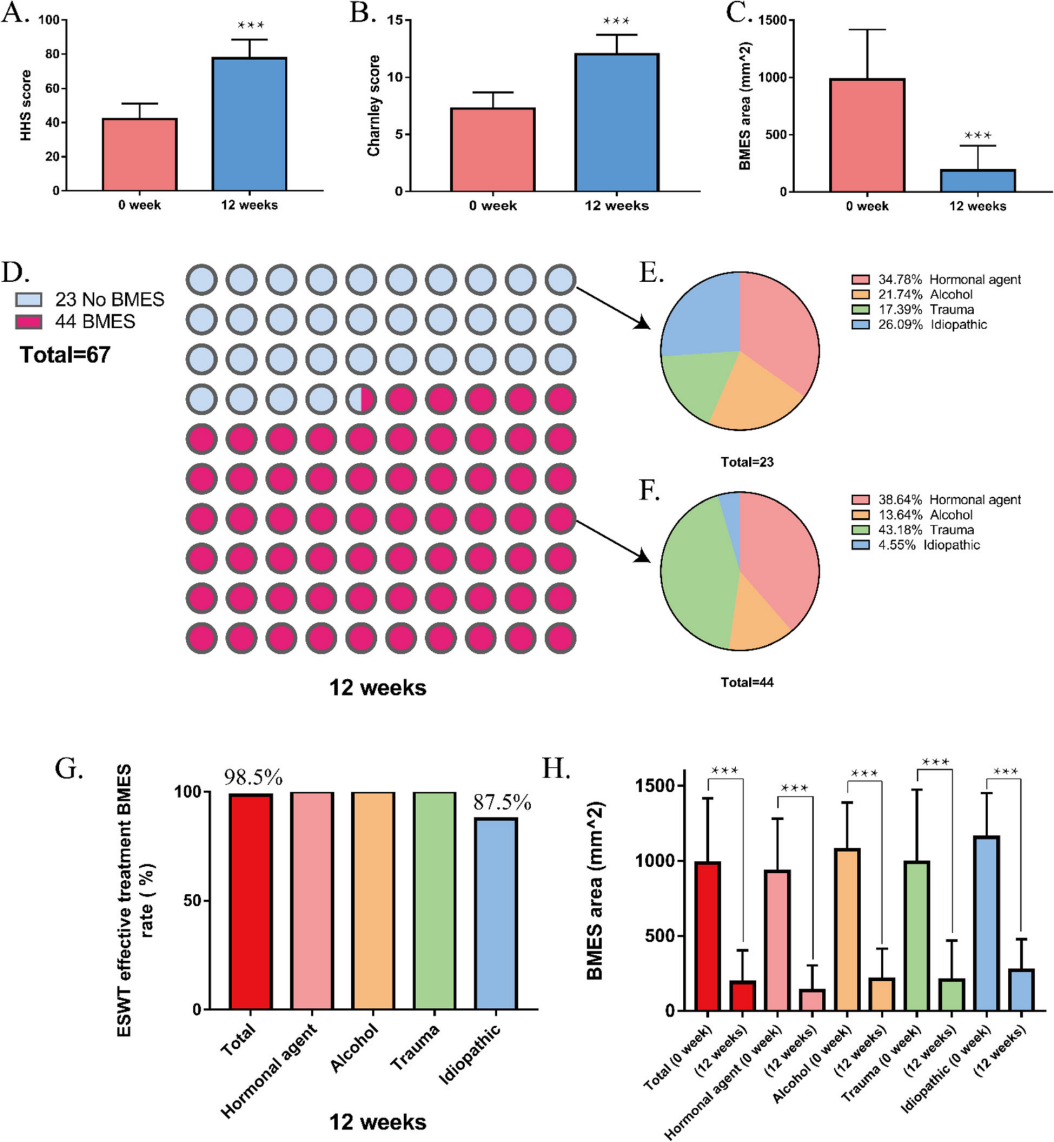
Extracorporeal shockwave therapy (ESWT) evaluation. **a** Harris hip score. **b** Charnley score. **c** Area of bone marrow edema. **d** Bone marrow edema syndrome (BMES) number after 12 weeks of ESWT treatment. **e** No BMES etiology. **f** BMES etiology. **g** ESWT effective treatment rate for BMES (%). **h** Area of bone marrow edema. ***P < 0.01

HHS improved from 42.2 ± 9.1 points before treatment to 77.7 ± 10.8 points (Table 2, Fig. 3a), with a significant difference before and after treatment (*P* < 0.0001). The Charnley score improved from 7.3 ± 1.4 points before treatment to 12.0 ± 1.7 points (Table 2, Fig. 3b), with a significant difference before and after treatment (*P* < 0.0001). The area of BMES decreased from 984.6 ± 433.2 mm^2^ before treatment to 189.7 ± 214.4 mm^2^ (Table 2, Fig. 3c), and there was a significant difference before and after treatment (*P* < 0.0001). After 12 weeks of ESWT, BMES disappeared in 23 patients (hormonal agent 34.78%, alcohol 21.74%, trauma 17.39%, idiopathic 26.09%; Fig. 3e), and 44 patients still had BMES (hormonal agent 38.64%, alcohol 13.64%, trauma 43.18%, idiopathic 4.55%; Fig. 3f) (Fig. 3d). The efficacy of ESWT in treating BMES was 100% in the hormonal agent, alcohol, and trauma groups each and 87.5% in the idiopathic group. The overall efficacy was 98.5% (Fig. 3g). After ESWT, the area of bone marrow edema caused by the different etiologies decreased significantly (*P* < 0.0001, Fig. 3h).

**Fig. 4:**
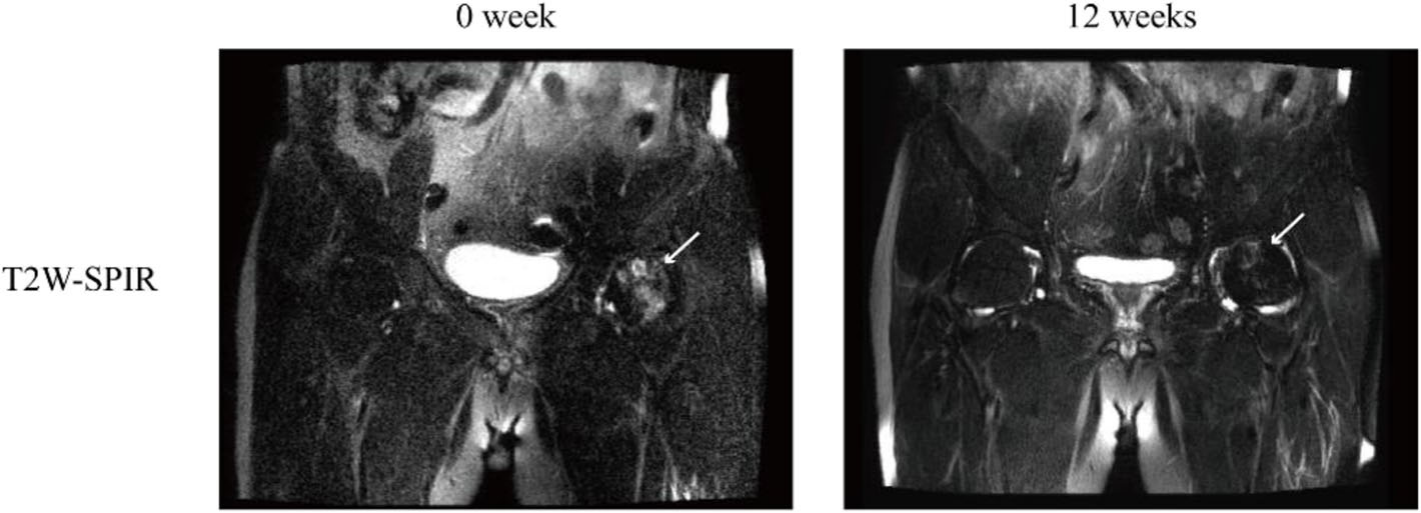
Cases of bone marrow edema syndrome affecting the femoral head after extracorporeal shockwave therapy

After 12 cycles of focused ESWT treatment, BMES improved significantly, and the area of bone marrow edema decreased significantly (Fig. 4). The patients’ pain significantly reduced, and the joint function significantly improved (Table 2).

## Discussion

BMES of the hip is usually a self-limiting disease that improves within 3 to 9 months without treatment. The current treatment of BMES mainly includes non-surgical treatment and surgical treatment [27]. The choice of treatment for BMES caused by ONFH should be based on comprehensive consideration of the stage, type, age, occupation, and compliance with hip preservation surgery; hospital conditions; physician’s skills; and the condition of the individual, with strict adherence to the surgical indications for BMES caused by ONFH and consideration of its traumatic and related side effects, for example, the total failure rate of treatment in patients with osteonecrosis undergoing total hip replacement is four times that in patients with osteoarthritis undergoing the same surgery [28]. Thus, non-surgical treatment, especially physical therapy, stands out due to its high safety, low treatment cost, and low trauma [29]. Among them, ESWT is gradually being accepted by people, with good analgesia, high safety, good tolerance, high compliance, and better treatment of BMES [30]; in addition, shock waves can significantly reduce both BMES symptoms and pain within 3 months. This study provides medical evidence for the efficacy of ESWT for BMES in a short period of time.

The appearance of BMES is a special phenomenon in the progress of necrosis, which is considered to be a sign of potential progression to advanced femoral head necrosis, and when BMES is observed, it is necessary to carefully check for femoral head necrosis [13]. Meier et al. believe that once BMES appears, it means that femoral head necrosis has entered ARCO stage III [31]. Clinically, it has been observed that BMES may continue to exist for a long time after ONFH collapse. The reason may be related to the instability of the femoral head after ONFH collapse. Previous studies have shown that the occurrence of femoral head necrosis is mainly related to mechanical factors. After the collapse, the stability in the femoral head is obviously lost, and the instability in the femoral head produces stress concentration, which results in the presence and long-term existence of BMES [8]. The presence of BMES also provides a certain reference for the treatment of ONFH after a collapse. The unstable femoral head requires proper braking rest, physical therapy, and necessary surgical intervention to create a stable mechanical environment for the repair of necrosis. In ONFH without BMES, this situation can allow patients to perform the necessary functional exercise activities. Therefore, the presence or absence of BMES in the femoral head after collapse can be used as an evaluation standard for the effect of treatment of ONFH, and it is also the clinical imaging basis for further treatment. However, this concept needs to be combined with long-term clinical follow-up and evaluation to reveal its existence.

BMES can be divided into congestive, tumorigenic, traumatic, or vasogenic by different mechanisms [32]. Congestive BMES secondary to ONFH caused by drugs, such as steroids, is usually caused by vascular occlusion [33]. The mechanism of the therapeutic effect of ESWT may be due to the shock wave’s dilation of blood vessels and angiogenesis [17]. Because ESWT has a powerful ability to generate pressure waves and cavitation, it can improve blood microcirculation [34]. To a certain extent, it can explain the efficacy of ESWT in treating BMES due to hormonal agent in this study. In congestive BMES secondary to ONFH caused by trauma, traumatic trabecular bone fracture leads to bleeding, and interstitial fluid enters the extracellular space, causing BMES. ESWT can promote bone tissue repair and regeneration and accelerate the repair process [35]. To a certain extent, it can explain the efficacy of ESWT in treating BMES caused by trauma in this study. Ethanol can significantly reduce the proliferation and osteogenic differentiation of bone marrow mesenchymal stem cells [36], which is one of the pathogenesis factors of ONFH. ESWT significantly promoted bone morphogenic protein (BMP)-2, BMP-3, BMP-4, and BMP-7 mRNA expression in the callus [5], and BMP is essential for fracture healing. To some extent, it can explain the efficacy of ESWT in treating BMES due to alcohol in this study (Fig. 3g, h). We believe that ESWT has a therapeutic effect because it breaks the vicious circle of bone marrow edema and necrosis. By improving BMES, ESWT can reduce intraosseous pressure and thus alleviate the pain caused by ONFH. By causing trabecular fractures to promote bone healing, necrotic bone tissue re-enters the cycle of bone reconstruction.

The purpose of BMES treatment is to shorten the clinical course and reduce pain. Related clinical studies have shown that anti-tuberculosis drugs, prednisone, calcitonin, and sympathectomy of the lumbar spine confer no significant treatment benefits in patients with BMES. Bupivacaine and nifedipine can alleviate the patient’s pain to a certain extent but cannot affect the progress of the disease [37]. The use of pulsed electromagnetic fields for treating BMES has been reported. In a study involving six patients with talus BMES, pulsed electromagnetic field stimulation was used daily for 30 days. In five patients, BMES improved after 1 month of treatment and completely resolved within 3 months [38]. However, the study had a small number of patients, and 30 days of continuous treatment might have resulted in poor patient compliance, highlighting the need for stronger clinical evidence. The average clinical recovery period of the eight patients treated with alendronate oral therapy was 6 months [39], whereas the average clinical recovery period of the six patients treated using calcitonin was 4.7 months [40]; the duration of recovery following core decompression therapy was between 1 week and 8 months (median: 1.5–2 months) [40]. However, although core decompression is the surgical gold standard, achieving immediate decompression and hip pain relief after surgery, this is an invasive treatment; possible side effects include infection, hematoma, etc. In our study, the efficacy of ESWT for BMES was 98.5% at 3 months; the patients showed higher compliance, and no side effects were observed.

Regarding the use of ESWT for treating BMES, there have been reports of satisfactory treatment results. Leilei et al. used ESWT to treat 34 patients with hip BMES and found that compared with that before treatment, hip pain was relieved to varying degrees in the first and third months after treatment; HHS was significantly improved, and visual analogy score was significantly reduced (*P* < 0.05). MRI results showed that the diffuse BMES of the femur disappeared completely [30]. D’Agostino et al. treated 20 patients with ONFH and BMES through ESWT [41]. The study found that compared with before treatment, in the sixth month after treatment intervention, hip pain was significantly relieved, hip function was significantly improved, and the average area of bone marrow edema decreased from 981.9 ± 453.2 mm^2^ before treatment to 107.8 ± 248.1 mm^2^ after treatment. A study involving 36 patients with avascular necrosis of femoral head showed that ESWT can help control the progression of non-vascular necrosis areas in patients with ARCO stages I and II and significantly reduce pain [42]. ESWT is an effective physical therapy that can quickly relieve pain, improve function and help normalize the area of bone marrow edema. In this study, although the overall cure rate for BMES was only 34.3%, ESWT significantly reduced the area of bone marrow edema, and the efficacy of ESWT for BMES was 98.5% (Fig. 3g). The area of BMES decreased from 984.6 ± 433.2 mm^2^ before treatment to 189.7 ± 214.4 mm^2^ (Table 2, Fig. 3c), and there was a significant difference before and after treatment (*P* < 0.0001). Our research results support the above evidence.

### Strengths and limitations of this study

Given that research on ESWT for treating ONFH caused by BMES is insufficient, this article provides a reference for its clinical application. In addition, the cure rate of ESWT for BMES caused by ONFH of different etiologies was discussed. The advantage of this research lies in its relatively easy operation and repeatability. While this study has achieved a good therapeutic effect, we need to acknowledge the following limitations of the study: the number of patients with BMES due to ONFH caused by different etiologies was small; there was no control group, and the follow-up time was short. Therefore, we look forward to adopting a large sample size, long-term follow-up, and a standardized multicenter randomized controlled trial in the future for further research. The mechanism by which ESWT exerts a therapeutic effect should be further revealed.

## Conclusions

ONFH has a multifactorial etiology, and the causes of BMES also vary. This study demonstrated the effectiveness of ESWT in treating BMES caused by ONFH by providing pain relief and enabling functional recovery. Given that the efficacy of ESWT for BMES was 98.5% and that ESWT is easy to perform, inexpensive, safe, and related to good tolerance and high compliance, our results further validate ESWT as the treatment of choice for BMES.

## Data Availability

The detailed data and materials of this study were available from the corresponding author through emails on reasonable request.

## Abbreviations

ARCO: Association Research Circulation Osseous
BMES: Bone marrow edema syndrome
ESWT: Extracorporeal shock wave therapy
HHS: Harris hip score
MRI: Magnetic resonance imaging
ONFH: Osteonecrosis of the femoral head

## References

[1] Maffulli G, Hemmings S, Maffulli N (2014). Assessment of the effectiveness of extracorporeal shock wave therapy (ESWT) for soft tissue injuries (ASSERT): an online database protocol. Transl Med UniSa..

[2] Maffulli G, Padulo J, Iuliano E, Furia J, Rompe J, Maffulli N (2018). Extracorporeal shock wave therapy in the management of insertional plantar fasciitis: the ASSERT database. Muscles Ligaments Tendons J.

[3] Mont MA, Zywiel MG, Marker DR, McGrath MS, Delanois RE (2010). The natural history of untreated asymptomatic osteonecrosis of the femoral head: a systematic literature review. J Bone Joint Surg Am.

[4] Gao F, Sun W, Li Z, Guo W, Kush N, Ozaki K (2015). Intractable bone marrow edema syndrome of the hip. Orthopedics..

[5] Ghasemi RA, Sadeghi S, Rahimee N, Tahmasebi M (2019). Technologies in the treatment of bone marrow edema syndrome. Orthop Clin North Am..

[6] Jianchuan W, Lei Y, Benjie W, Dewei Z (2015). Study on correlation between bone marrow edema, stage of necrosis and area ratio of necrosis with the hip pain grading in nontraumatic osteonecrosis of the femoral head. Open Med (Wars).

[7] O'Hare A, Shortt C, Napier N, Eustace SJ (2006). Bone marrow edema: patterns and clinical implications. Semin Musculoskelet Radiol..

[8] Hynes JP, Hughes N, Cunningham P, Kavanagh EC, Eustace SJ (2019). Whole-body MRI of bone marrow: a review. J Magn Reson Imaging..

[9] Kubo T, Yamamoto T, Inoue S, Horii M, Ueshima K, Iwamoto Y (2000). Histological findings of bone marrow edema pattern on MRI in osteonecrosis of the femoral head. J Orthop Sci..

[10] Akisue T, Matsumoto K, Tsumura N, Fujita I, Yamamoto T, Yoshiya S (2003). Bone marrow edema syndrome associated with uterine myoma: a case report. Clin Orthop Relat Res.

[11] Koo KH, Ahn IO, Kim R, Song HR, Jeong ST, Na JB (1999). Bone marrow edema and associated pain in early stage osteonecrosis of the femoral head: prospective study with serial MR images. Radiology..

[12] Chan W, Liu YJ, Huang GS, Jiang CC, Huang S, Chang YC (2002). MRI of joint fluid in femoral head osteonecrosis. Skeletal Radiol.

[13] Iida S, Harada Y, Shimizu K, Sakamoto M, Moriya H (2000). Correlation between bone marrow edema and collapse of the femoral head in steroid-induced osteonecrosis. AJR Am J Roentgenol.

[14] Liao CD, Xie GM, Tsauo JY, Chen HC, Liou TH (2018). Efficacy of extracorporeal shock wave therapy for knee tendinopathies and other soft tissue disorders: a meta-analysis of randomized controlled trials. BMC Musculoskelet Disord..

[15] Maffulli G, Padulo J, Iuliano E, Saxena A, Rompe J, Maffulli N (2019). Extracorporeal shock wave therapy in the management of midsubstance Achilles tendinopathy: the ASSERT database. Muscles Ligaments Tendons J.

[16] Maffulli G, Iuliano E, Padulo J, Gerdesmeyer L, Rompe J, Maffulli N (2019). Extracorporeal shock wave therapy in the treatment of calcific tendinopathy of the shoulder: the ASSERT database. Muscles Ligaments Tendons J.

[17] Ma HZ, Zhou DS, Li D, Zhang W, Zeng BF (2017). A histomorphometric study of necrotic femoral head in rabbits treated with extracorporeal shock waves. J Phys Ther Sci..

[18] Ochiai N, Ohtori S, Sasho T, Nakagawa K, Takahashi K, Takahashi N (2007). Extracorporeal shock wave therapy improves motor dysfunction and pain originating from knee osteoarthritis in rats. Osteoarthritis Cartilage..

[19] Holfeld J, Tepeköylü C, Kozaryn R, Urbschat A, Zacharowski K, Grimm M (2014). Shockwave therapy differentially stimulates endothelial cells: implications on the control of inflammation via toll-like receptor 3. Inflammation..

[20] Sadile F, Bernasconi A, Russo S, Maffulli N (2016). Core decompression versus other joint preserving treatments for osteonecrosis of the femoral head: a meta-analysis. Br Med Bull..

[21] Vitali M, Naim Rodriguez N, Pedretti A, Drossinos A, Pironti P, Di Carlo G (2018). Bone marrow edema syndrome of the medial femoral condyle treated with extracorporeal shock wave therapy: a clinical and MRI retrospective comparative study. Arch Phys Med Rehabil..

[22] Gardeniers JWM, Gosling-Gardeniers AC, Rijnen WHC, Koo KH, Mont MA, Jones LC (2014). The ARCO staging system: generation and evolution since 1991. Osteonecrosis.

[23] Yadav H, Khanduri S, Yadav P, Pandey S, Khan S (2020). Diagnostic accuracy of dual energy CT in the assessment of traumatic bone marrow edema of lower limb and its correlation with MRI. Indian J Radiol Imaging.

[24] Cunningham G, Lädermann A, Denard PJ, Kherad O, Burkhart SS (2015). Correlation between American shoulder and elbow surgeons and single assessment numerical evaluation score after rotator cuff or SLAP repair. Arthroscopy..

[25] Wall PDH, Hossain M, Beard DJ, Murray DW, Andrew JW (2013). The effect of locomotion on the outcome following total hip arthroplasty. Hip Int.

[26] Machin D, Campbell MJ, Tan SB, Tan SH (2008). Sample size tables for clinical studies S. 3rd ed. Wiley-Blackwell.

[27] Hofmann S (2005). The painful bone marrow edema syndrome of the hip joint. Wien Klin Wochenschr..

[28] Cornell CN, Salvati EA, Pellicci PM (1985). Long-term follow-up of total hip replacement in patients with osteonecrosis. Orthop Clin North Am..

[29] Chughtai M, Piuzzi NS, Khlopas A, Jones LC, Goodman SB, Mont MA (2017). An evidence-based guide to the treatment of osteonecrosis of the femoral head. Bone Joint J..

[30] Zhang L, Cui Y, Liang D, Guan J, Liu Y, Chen X (2020). High-energy focused extracorporeal shock wave therapy for bone marrow edema syndrome of the hip: a retrospective study. Medicine.

[31] Meier R, Kraus TM, Schaeffeler C, Torka S, Schlitter AM, Specht K (2014). Bone marrow oedema on MR imaging indicates ARCO stage 3 disease in patients with AVN of the femoral head. Eur Radiol..

[32] Koff MF, Burge AJ, Potter HG (2020). Clinical magnetic resonance imaging of arthroplasty at 1.5 T. J Orthop Res..

[33] Wang GJ, Dughman SS, Reger SI, Stamp WG (1985). The effect of core decompression on femoral head blood flow in steroid-induced avascular necrosis of the femoral head. J Bone Joint Surg Am..

[34] Wang S, Yin C, Han X, Guo A, Chen X, Liu S (2019). Improved healing of diabetic foot ulcer upon oxygenation therapeutics through oxygen-loading nanoperfluorocarbon triggered by radial extracorporeal shock wave. Oxid Med Cell Longev.

[35] Zissler A, Steinbacher P, Zimmermann R, Pittner S, Stoiber W, Bathke AC (2017). Extracorporeal shock wave therapy accelerates regeneration after acute skeletal muscle injury. Am J Sports Med.

[36] Yu H, Zhu D, Liu P, Yang Q, Gao J, Huang Y (2020). Osthole stimulates bone formation, drives vascularization and retards adipogenesis to alleviate alcohol-induced osteonecrosis of the femoral head. J Cell Mol Med..

[37] Wang FS, Yang KD, Kuo YR, Wang CJ, Sheen-Chen SM, Huang HC (2003). Temporal and spatial expression of bone morphogenetic proteins in extracorporeal shock wave-promoted healing of segmental defect. Bone..

[38] Martinelli N, Bianchi A, Sartorelli E, Dondi A, Bonifacini C, Malerba F (2015). Treatment of bone marrow edema of the talus with pulsed electromagnetic fields: outcomes in six patients. J Am Podiatr Med Assoc..

[39] Hofmann S, Engel A, Neuhold A, Leder K, Kramer J, Plenk H (1993). Bone-marrow oedema syndrome and transient osteoporosis of the hip. An MRI-controlled study of treatment by core decompression. J Bone Joint Surg Br.

[40] Asadipooya K, Graves L, Greene LW (2017). Transient osteoporosis of the hip: review of the literature. Osteoporos Int..

[41] D’Agostino C, Romeo P, Lavanga V, Pisani S, Sansone V (2014). Effectiveness of extracorporeal shock wave therapy in bone marrow edema syndrome of the hip. Rheumatol Int..

[42] Vulpiani MC, Vetrano M, Trischitta D, Scarcello L, Chizzi F, Argento G (2012). Extracorporeal shock wave therapy in early osteonecrosis of the femoral head: prospective clinical study with long-term follow-up. Arch Orthop Trauma Surg..

